# BoostMEC: predicting CRISPR-Cas9 cleavage efficiency through boosting models

**DOI:** 10.1186/s12859-022-04998-z

**Published:** 2022-10-26

**Authors:** Oscar A. Zarate, Yiben Yang, Xiaozhong Wang, Ji-Ping Wang

**Affiliations:** 1 Department of Statistics and Data Science, Northwestern University, Evanston, IL USA; 2 Department of Molecular BioSciences, Northwestern University, Evanston, IL USA

**Keywords:** CRISPR-Cas9, sgRNA, Feature engineering, Machine learning, Regression trees, LightGBM, Interpretability

## Abstract

**Background:**

In the CRISPR-Cas9 system, the efficiency of genetic modifications has been found to vary depending on the single guide RNA (sgRNA) used. A variety of sgRNA properties have been found to be predictive of CRISPR cleavage efficiency, including the position-specific sequence composition of sgRNAs, global sgRNA sequence properties, and thermodynamic features. While prevalent existing deep learning-based approaches provide competitive prediction accuracy, a more interpretable model is desirable to help understand how different features may contribute to CRISPR-Cas9 cleavage efficiency.

**Results:**

We propose a gradient boosting approach, utilizing LightGBM to develop an integrated tool, BoostMEC (Boosting Model for Efficient CRISPR), for the prediction of wild-type CRISPR-Cas9 editing efficiency. We benchmark BoostMEC against 10 popular models on 13 external datasets and show its competitive performance.

**Conclusions:**

BoostMEC can provide state-of-the-art predictions of CRISPR-Cas9 cleavage efficiency for sgRNA design and selection. Relying on direct and derived sequence features of sgRNA sequences and based on conventional machine learning, BoostMEC maintains an advantage over other state-of-the-art CRISPR efficiency prediction models that are based on deep learning through its ability to produce more interpretable feature insights and predictions.

**Supplementary Information:**

The online version contains supplementary material available at 10.1186/s12859-022-04998-z.

## Background

The CRISPR-Cas9 system is a powerful tool for genetic engineering that can be programmed to target specific regions of a given genome [1–3]. CRISPR can be used to modify genomes for applications as varied as pathway analysis [4], drug screens [5], and gene therapy [6, 7]. The versatility of the technology comes from the programmability of the single guide RNA (sgRNA, or gRNA), an RNA sequence approximately 100 nucleotides (nt) long comprised of a spacer sequence of 20 nt and a scaffold sequence of approximately 80 nt. The spacer sequence can be engineered to match a 20 nt target DNA sequence in an organism of interest and designates where Cas9 will cut the genome. These 20 nt target regions must be followed by a short pattern in the target genome, typically 2–6 nucleotides long depending on the CRISPR-Cas system used, known as a protospacer adjacent motif (PAM). In the CRISPR-Cas9 system, the PAM is a 3 nt sequence, NGG, where N designates any of the 4 DNA nucleotides. Thus, genomic regions targeted for cleavage using the CRISPR-Cas9 system typically follow the pattern 5’-N20-NGG-3’, where the 20-nucleotide sequence specified by N20 is used to create a matching sgRNA and the NGG designates the typical 3-nucleotide PAM sequence used by Cas9.

In recent years, a variety of models have been created for the prediction of sgRNA on-target efficiency. Features are typically generated through one-hot encoding of sgRNA target region nucleotides and dinucleotides, obtaining k-mer counts for those regions, and through the generation of features based on thermodynamic and epigenetic properties of the sgRNAs and target regions. Model architecture choices are varied and have included linear regression [8, 9], binomial regression [8], SVMs [10–12], elastic net [13], boosted regression trees [14, 15], Bayesian ridge regression [16], multi-step models [17–19], convolutional neural networks (CNN) [20–23], and recurrent neural networks (RNN) [24].

A review by Haeussler et al. [25] compared the performance of various CRISPR prediction algorithms and found that cross-dataset performance strongly depended on whether sgRNAs were produced within cells via a U6 promoter or in vitro via a T7 promoter and then injected. Furthermore, a recent study evaluated eight regression models by training them on five different CRISPR screen datasets from different species and evaluating their performance on their own test sets and that of the other species [16]. The authors selected the best model for each species for evaluation on the other species’ datasets. They showed that the best model varied across datasets; the best models included random forests, the lasso, gradient boosted regression trees, and Bayesian ridge regression.

While deep learning-based approaches achieve considerable success on this problem, they often lack interpretability—insights into how specific predictions were made. In many areas of application, but particularly in health-adjacent fields, the use of black-box models without insights into their decision-making process can limit their trustworthiness and hence, further adoption and use of machine learning [26]. In contrast, methods based on conventional statistical or machine learning methods, often shown to have less competitive performance, are continuing to be explored for their interpretable properties. For example, in one recent study, Konstantakos and coauthors [8] developed a prediction tool based on binomial and linear regression, CRISPRedict, which achieves competitive performance compared to other recent tools, but with the added benefit of model explainability and interpretable predictions. In this paper, we contribute a novel tool termed BoostMEC for CRISPR efficiency prediction. We show that BoostMEC features prediction interpretation capabilities while achieving state-of-the-art performance.

## Results

### Data

Our training data is derived from sgRNA efficiency datasets from two studies: Kim et al. [21] and Xiang et al. [22], which we combined in the same manner that Xiang et al. outlined for the training of their prediction tool CRISPRon. Both studies produced high-throughput sgRNA efficiency data for HEK293T cells, a human kidney cell line, using sgRNA and synthetic target region pairs that were delivered via lentivirus. Lentiviral integration of the expanded target region reduces the impact of chromatin accessibility on measured CRISPR activity, hence the indel rates produced by these screens can better reveal how sgRNA efficiency is impacted by sequence features [24, 27–29]. From Kim et al. [21], we combined the HT_Cas9_Train and HT_Cas9_Test datasets, which contained 12,832 and 542 sgRNAs, respectively, to produce one dataset with 13,359 sgRNAs after averaging duplicates between the datasets. From Xiang et al. [22], we followed the processing steps specified in the paper including selecting sgRNAs with at least 200 reads from their Day 8 and Day 10 doxycycline-negative datasets and averaging the intersection to obtain 10,592 unique sgRNAs. We found 49 overlapping sgRNAs between the Kim and Xiang datasets and utilized linear regression on this set of sgRNAs to produce a normalization model to adjust the Xiang data to the scale of the Kim data. We then combined the Kim and rescaled Xiang datasets into one large training dataset composed of 23,902 unique sgRNAs (the efficiency value was averaged for guides shared between the datasets as was done in Xiang et al. [22]) which is to be termed the Kim-Xiang dataset henceforth.

### Overall model performance

We tuned the LightGBM hyperparameters for BoostMEC using tenfold cross-validation and Bayesian hyperparameter optimization (Methods). To quantify model performance, we follow most existing work by using Spearman correlation, keeping the ranking nature of the task in focus, and accounting for the non-linearity commonly observed in predicted efficiency scores (Figure S1 in Additional File 1). The final parameter configuration achieved an average Spearman correlation of 0.78 on the validation folds, and the full Kim-Xiang dataset was used to train the final BoostMEC model. To assess the performance of BoostMEC more rigorously, we selected 13 external test datasets adapted from the repository for the study by Haeussler et al. [25] (https://github.com/maximilianh/crisporPaper; the repository datasets are named in parentheses below for convenience of discussion). These datasets include U6 promoter CRISPR efficiency datasets created from HL60 cells (xu2015TrainHl60) [12, 13], KBM-7 cells (xu2015TrainKbm7) [12, 13], HEK293T cells (chari2015Train293T) [10], HeLa cells (hart2016-HelaLib1Avg and hart2016-HelaLib2Avg) [30], HCT116 cells (hart2016-Hct1162lib1Avg) [30], RPE-1 cells (hart2016-Rpe1Avg) [30], a dataset derived from MOLM13, NB4 and TF1 cells (doench2014-Hs) [31], EL4 cells (doench2014-Mm) [31], and A375 cells (doench2016azd_hg19) [14, 15]. Also included were three T7 promoter CRISPR efficiency datasets created from zebrafish embryos (morenoMateos2015, gagnon2014, varshney2015) [9, 32, 33]. Note that for xu2015TrainHl60 and xu2015TrainKbm7, efficiency was measured via log2 fold change in negative selection screens (lower values indicate stronger efficiency); hence, we reversed the reported efficiency scores during model evaluation for consistency with the other datasets.

**Fig. 1:**
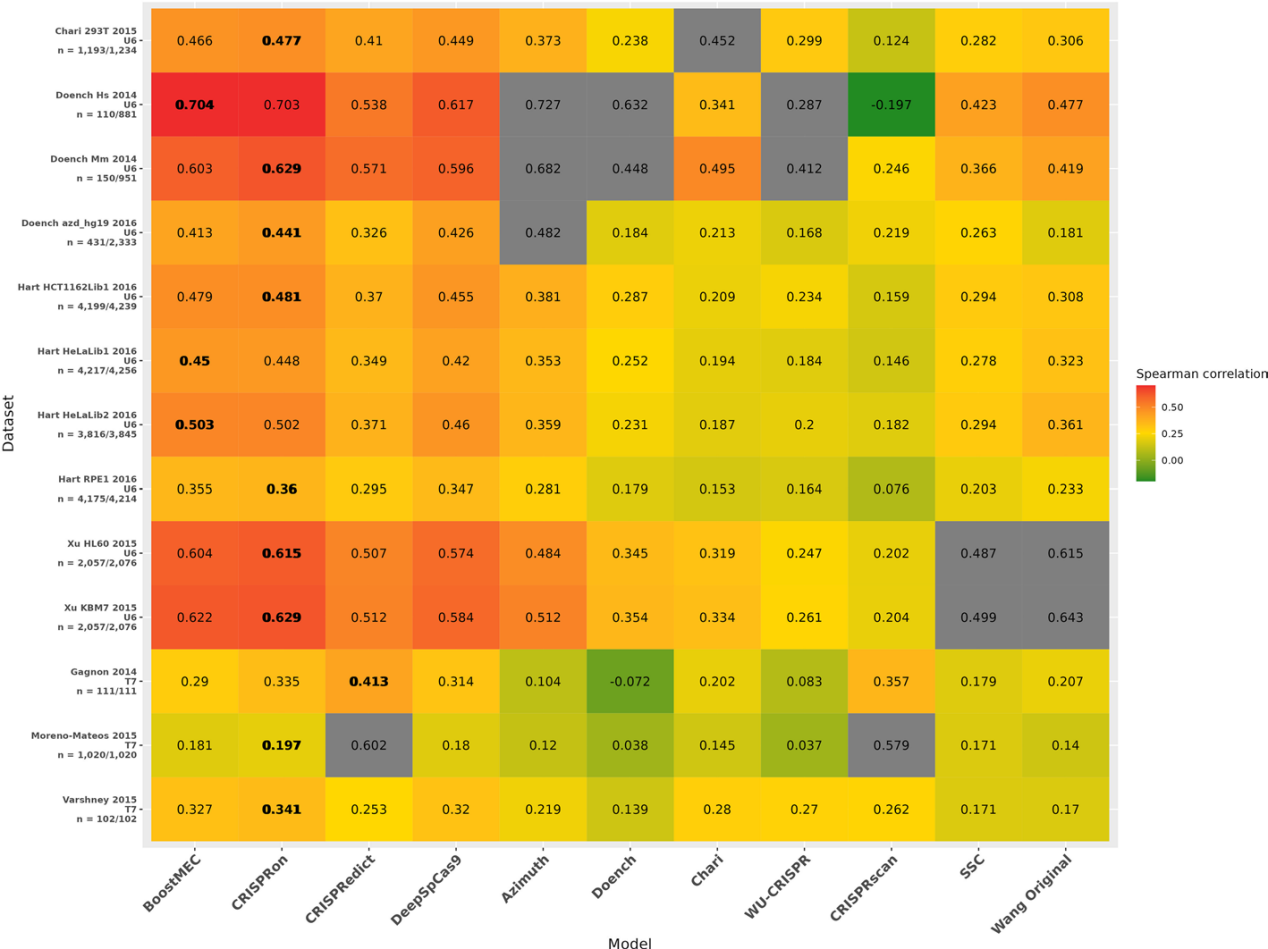
Comparison of model performance, measured through Spearman correlation, for 11 CRISPR-Cas9 cleavage efficiency prediction methods. Under comparison are the proposed method BoostMEC and other 10 methods, among which, predictions for CRISPRon, CRISPRedict, and DeepSpCas9 were obtained by utilizing the software made available by the authors of each study, and the rest were adapted from the study by Haeussler et al. [25]. The details of the 13 external datasets were described in the text. Cells grayed out indicate that dataset was used in the training of that method, and thus not included in the comparison. Cells with bold figures indicate that method achieved maximum nominal Spearman correlation among the 11 methods for that external dataset under testing

We compared BoostMEC with 10 other competing models, including CRISPRon (CNN) [22], CRISPRedict (2 separate linear models, each optimized for U6 or T7 promoters) [8], DeepSpCas9 (CNN) [21], Azimuth (boosted regression trees) [14, 15], and others [9, 10, 12, 13, 31, 34] utilized in Haeussler et al. [25]. Predictions for CRISPRon, CRISPRedict, and DeepSpCas9 were obtained by utilizing the software made available by the authors of each study. All other predictions were adapted from the study by Haeussler et al. [25]. We will focus more on the comparison between BoostMEC and more recent approaches including CRISPRon, DeepSpCas9, and CRISPRedict, as they have been shown to have the most competitive performance. For more rigor, we removed the sgRNAs in these test datasets if they overlapped with the combined Kim-Xiang training dataset, which was used in part or whole for the training of BoostMEC, CRISPRon, DeepSpCas9, and CRISPRedict (CRISPRedict also used data from Moreno-Mateos et al. [9] for training their T7 model). The results are presented in Fig. 1.

**Fig. 2:**
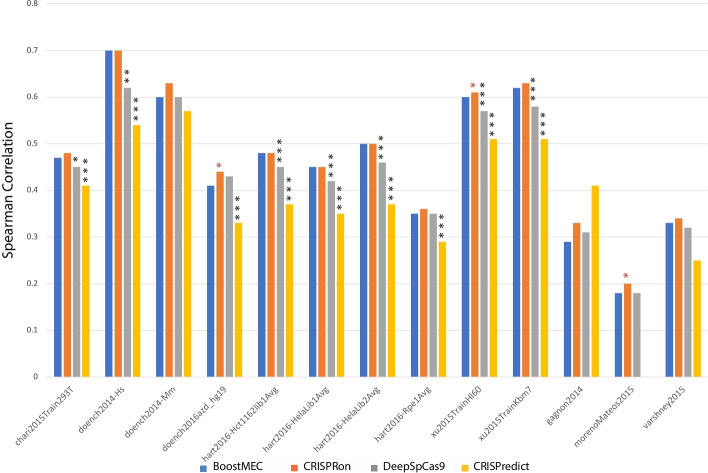
Significance test of performance difference between BoostMEC and CRISPRon, DeepSpCas9, and CRISPRedict. Each bar represents the Spearman correlation obtained in the 13 external testing datasets for the four methods. Pairwise Steiger’s tests were performed for BoostMEC with CRISPRon, DeepSpCas9, and CRISPRedict. Asterisks on top of the bars of CRISPRon, DeepSpCas9, and CRISPRedict indicate the test significance of the *p*-value (*: *p*-value < 0.05, **: *p*-value < 0.005 and ***: *p*-value < 0.0005). Red asterisks indicate the Spearman correlation from the method under consideration is significantly higher than BoostMEC whereas black asterisks indicate the opposite. Note: The morenoMateos2015 dataset was used in the training of CRISPRedict, thus it was dropped in the comparison between BoostMEC and CRISPRedict

Among the 11 methods, CRISPRon, BoostMEC, DeepSpCas9, and CRISPRedict show pronouncedly better performance than the rest. Among these four, CRISPRon is the overall winner and achieved the highest nominal Spearman correlation in 9 test datasets, whereas BoostMEC, CRISPRedict and DeepSpCas9 excelled in 3, 1 and 0, respectively. It should be noted that CRISPRedict has two variants, one trained on U6 promoter data, and the other separately trained on T7 promoter data. Thus, its relatively stronger performance in T7 promoter data is not surprising compared to BoostMEC, CRISPRon and DeepSpCas9, as the latter three were all trained based on U6 promoter data. To test whether the observed differences are significant, we performed pairwise Steiger’s tests on the Spearman correlation values between BoostMEC and the other three methods using the *psych* R package; the results are presented in Fig. 2. The Spearman correlation of CRISPRon is significantly higher than BoostMEC at the 0.05 significance level in three datasets, namely: doench2016azd_hg19 (Spearman correlation 0.44 vs. 0.41, *p*-value = 0.010), xu2015TrainHl60 (0.61 vs. 0.60, *p*-value = 0.016), and morenoMateos2015 (0.20 vs 0.18 *p*-value = 0.039), while BoostMEC surpasses CRISPRon in none. BoostMEC significantly outperforms DeepSpCas9 in 7 out of 13, and CRISPRedict in 9 out of the 12 external test datasets, respectively, while neither of the latter two surpass BoostMEC at the 0.05 significance level (note: morenoMateos2015 was used in training of CRISPRedict, thus it is not included in the comparison between BoostMEC and CRISPRedict). In summary, we conclude BoostMEC achieves the state-of-the-art prediction accuracy of CRISPR-Cas9 cleavage efficiency.

### Feature importance in cleavage efficiency

**Fig. 3:**
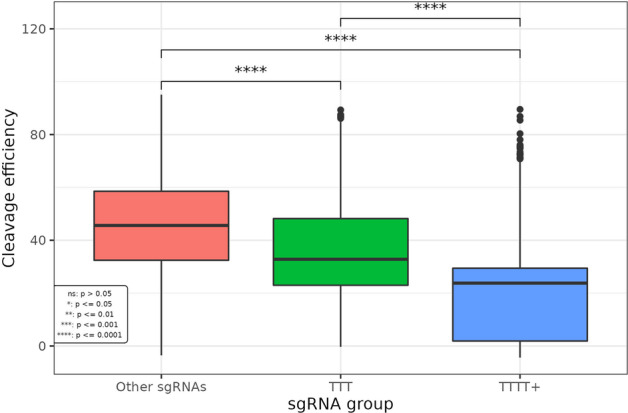
Comparison of observed CRISPR-Cas9 cleavage efficiency between sgRNAs with and without poly-T. From the combined Kim-Xiang training data, we identified 3,561 guides with a maximum poly-T length of 3 nt (TTT) and 1,276 guides with a poly-T sequence of length 4 or greater (TTTT +), with the longest sequence spanning 25 nt. The box plots present the distribution of the observed cleavage efficiency in the three groups and the statistical significance in pairwise comparisons from two-sample *t*-tests

Our features are all derived from expanded 30-mer target region sequences from the Kim-Xiang dataset, consisting of the 4 nt region upstream of the sgRNA, the 20 nt sgRNA-matching sequence, the 3 nt PAM, and the 3 nt region downstream of the PAM. In our early modeling work, we observed high feature importance values for the number of Ts, TTs, and TTTs across the 30-mer sequence. It has been reported that even poly-T sequences of length 3 are associated with decreased sgRNA activity [34–36]. We investigated the presence of poly-T sequences in the Kim-Xiang dataset and found 3,561 guides with a maximum poly-T length of 3 nt (TTT) and 1,276 guides with a poly-T sequence of length 4 or greater, with the longest sequence spanning 25 nt. We compare the cleavage efficiency of these three groups in Fig. 3, finding statistically significant differences between sgRNAs without these poly-T sequences (mean efficiency score 45.1) and the TTT and TTT + groups (mean efficiency scores 34.8 and 20.5, respectively, all pairwise *p*-values < 2 × 10^–16^), further supporting the hypothesis that RNA Pol III termination can occur with the presence of poly-T sequences of length 3. These results and one reviewer’s feedback prompted the inclusion of another set of sequenced-based features in our model: the number of poly-T sequences (contiguous stretches of 3 or more Ts) and length of the longest poly-T segment in the target region 30-mers.

**Fig. 4:**
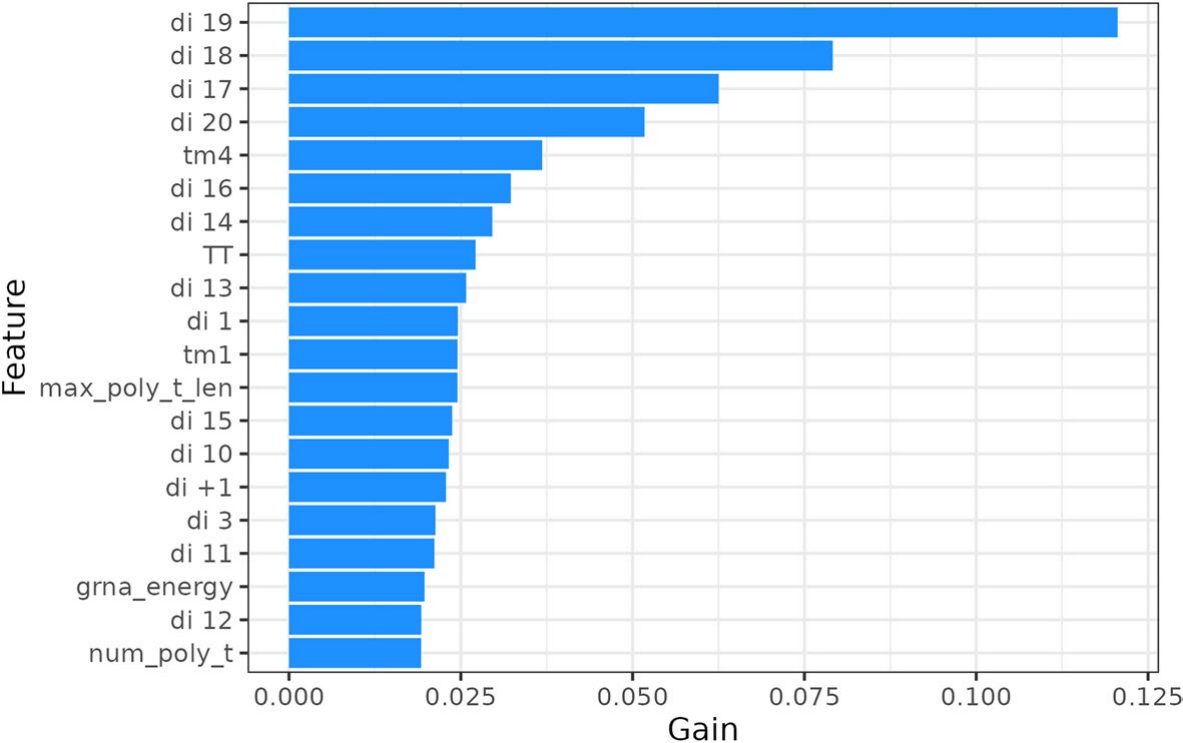
Feature importance chart for BoostMEC. The top 20 features for BoostMEC are listed in descending order of importance, showing the percentage gain contributed by all tree splits for each feature in the minimization of the LightGBM cost function under mean squared error loss

To investigate the role of each feature in BoostMEC, we computed the feature importance values. Each is characterized as the total gain, or improvement on the LightGBM objective function contributed by the given feature, which we further normalize to provide the proportion of gain contributed by each feature (the sum of all feature importance values adds up to 1). BoostMEC contains a total of 149 sequence or sequence-derived features (see Methods). The top 20 features are shown in Fig. 4, and the full list of feature importance values is available in Supplementary Table S1 (Additional File 1). In plots produced by BoostMEC, we denote position-specific mono and di-nucleotide k-mers by their position relative to the start of the combined sgRNA and PAM region; therefore, the positions of the region upstream of the sgRNA are indexed as −4 to −1, the sgRNA + PAM correspond to positions 1 through 23, and the region downstream of the PAM corresponds to positions + 1 to + 3. Furthermore, note that LightGBM allows direct use of categorical features without one-hot-encoding, therefore all position-specific mononucleotides and dinucleotides are only characterized by their position in the feature importance table.

Within the top 20 features ranked by importance, we observed position-specific features for positions 10 through + 1 and 1 through 3 of the sgRNA and surrounding expanded target region. Among those position-specific features, the highest ranked were concentrated on the 3’ end of the sgRNA and the first nucleotide of the PAM, including dinucleotide type at positions 19, 18, 17, and 20 (Fig. 4), consistent with findings from other studies [13, 24, 31]. Other features in the top 20 include 2 out of 4 melting temperature features, sgRNA free energy, and a number of poly-T-related features: number of TTs, maximum poly-T length, and number of poly-T segments in the 30-mer sequence. Melting temperature has previously been found to be a strong feature in feature importance investigations [14, 15, 18]. On the other hand, free energy has sometimes been found to be a useful discriminator in some studies [34], and not in others [9]. Interestingly, our feature importance results differ from those obtained by an alternative model created by Xiang et al. [22] trained using gradient boosting regression trees on the same training data sources; this is discussed further in the Discussion section.

### Model interpretation

**Fig. 5:**
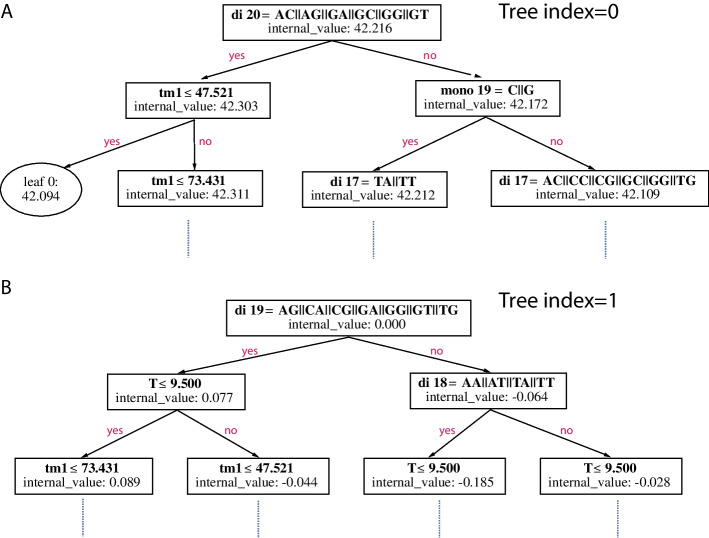
Schematic plot of regression trees from BoostMEC. BoostMEC consists of many sequencing trees. Plotted are the first (tree index = 0) and second (index = 1) regression trees from BoostMEC, truncated at depth = 3 for illustration purposes, i.e., only the branches/leaves in the first three hierarchical levels from the root are shown. The first tree plotted in **A** starts with the grand mean of the efficiency score (internal_value = 42.216) and splits based on whether the dinucleotide at position 20 is one of several values (di 20 = AC||AG||GA||GC||GG||GT) and so forth. The internal_value at each node represents the mean within each branch under the mean squared error loss function. An oval shape represents an end node whereas a rectangle represents an intermediate node that grows further. The second tree in **B** is constructed based on the residual values resulting from the first tree model (mean residual or internal_value = 0) and splits based on di19 and so forth. The full tree for 6A is available in Additional File 2, and the full tree for 6B is available in Additional File 3

The versatility of the LightGBM software package enables BoostMEC to produce a number of different visualizations that can aid in interpreting both the overall model and individual predictions. In addition to the standard feature importance values and plot previously discussed (Fig. 4 and Table S1 in Additional File 1), BoostMEC can provide more granular insights into its decisions by plotting out its component regression trees. As BoostMEC relies on gradient boosting, it consists of a sequence of regression trees. In Fig. 5, we show the first 2 regression trees of the model, up to a depth of 3 (manually edited to reduce tree width for illustration purposes; the full trees produced by the software are available in Additional Files 2 and 3). In the first tree, each node contains an “internal value,” which equals the mean of the group before that specific split (under the mean squared error loss function used here), or the value that would be assigned to a prediction should it stop at that node. When the tree stops growing, the end nodes become leaves. Thus, the internal value at the root node equals the grand mean of the efficiency scores of all sgRNA in the training data. At the start of subsequent trees, the residuals from previous aggregated tree models are used in the response for tree construction, and thus the root nodes always have an internal value of 0 under the mean squared error loss function.

In the root of the first tree, displayed in Fig. 5A, the importance of the 3’ end of the sgRNA and the first nucleotide of the NGG PAM region is immediately visible, with the model assigning a higher efficiency to sgRNAs with either a G in position 20, or AC and AG starting at position 20. LightGBM’s grouping strategy for categorical variables allows this initial split point to be highly informative and more interpretable than a series of binary splits, showing how a nucleotide and a subset of dinucleotides can be grouped together in a decision for the model.

In the second tree, shown in Fig. 5B, the dinucleotide at position 19 (di19), the most important overall feature as shown in Fig. 4, dictates the first split at the root node, followed by other prominent features such as dinucleotide at position 18 (di18), number of Ts in the 30-mer, and melting temperature for positions 1–21 (Tm1). The feature importance in Fig. 4 represents the proportion of error reduction in the LightGBM cost function due to all the splits contributed by a feature over all trees in the model.

### Prediction interpretation

**Fig. 6:**
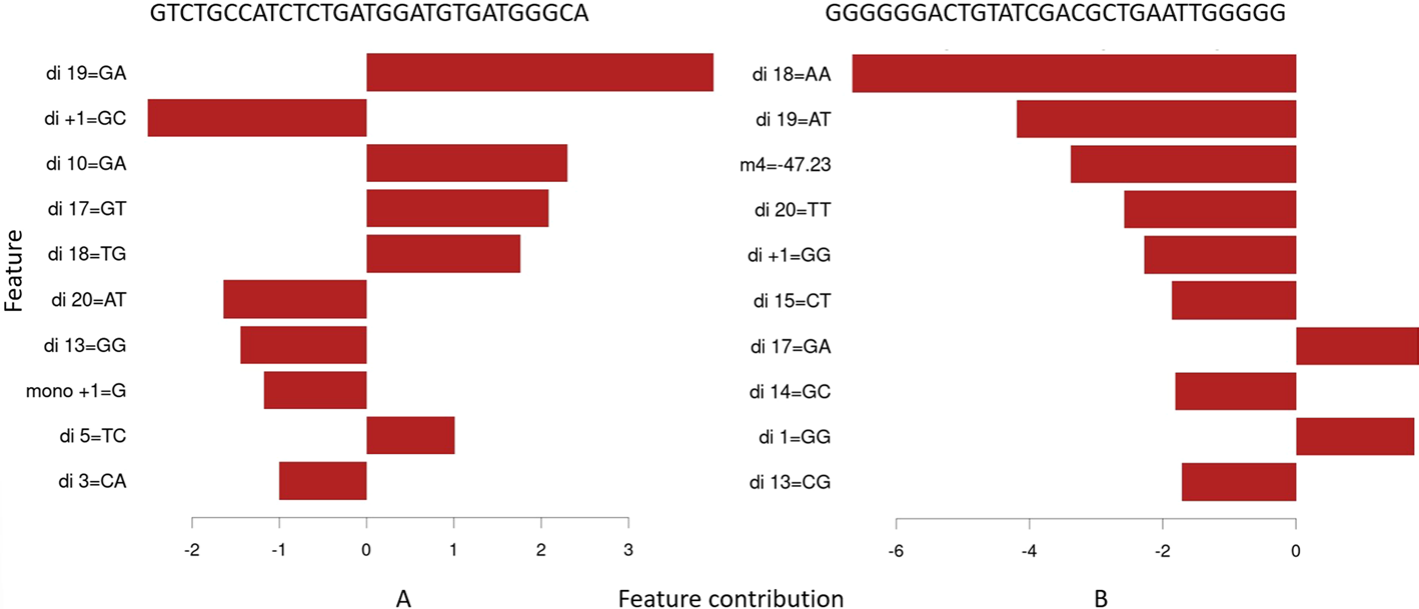
Feature contributions for individual sgRNA efficiency predictions. Two examples, **A** and **B**, show how the predicted efficiency scores were obtained under BoostMEC, using plots generated by LightGBM’s interprete function. The sequence for A comes from the doench2014-Hs dataset, and B’s sequence comes from the morenoMateos2015 dataset. In both examples, only the contributions of the top 10 features are plotted; the full sets of feature contribution values are available in Supplementary Tables S2 and S3. The predicted efficiency scores for these sequences are 49.5 and 28.4, respectively, and equal the grand mean of the efficiency score in the training data (42.2156) plus the sum of the individual feature contribution values for each sgRNA

For insights into the workings of specific predictions, BoostMEC also supports the generation of "interpretation plots" through LightGBM’s interprete function. These plots can highlight the total contributions, either positive or negative, that different features had for a specific prediction value. In Fig. 6, we exemplify this by showing the interpretation plots for two specific sequences, illustrating how individual sequence features contribute to the predicted efficiency. The first sequence, originating from doench2014-Hs, is GTCT-GCCATCTCTGATGGATGTGA-TGG-GCA (dashes separating the upstream region in positions -4 to -1, the sgRNA spacer region in positions 1–20, PAM positions 21–23, and downstream region + 1 to + 3) and the interpretation plot is shown in Fig. 6A. The second sequence, GGGG-GGACTGTATCGACGCTGAAT-TGG-GGG, is from morenoMateos2015 with the interpretation plot shown in Fig. 6B. The interpretation plots in Fig. 6 show the top 10 features that contribute to the predicted efficiency score for each sequence (the full set of sequence feature contributions can be found in Supplementary Tables S2 and S3 in Additional File 1). The predicted efficiency scores, 49.53 and 28.42 respectively, equal the sum of all feature contributions and the root node value for the first tree in the model (42.2156, the grand mean of the training data). These feature contributions provide a clear picture into how sequence features can dramatically impact sgRNA cleavage efficiency. For example, the dinucleotide at position 19 (di19), GA, AT in the two cases respectively, again shows up as the first and second important feature in the two cases, but impacting the efficiency score in opposite ways (scores are 3.98 vs. -4.2), as does another top feature di18 (TG vs. AA with scores 1.76 vs. -6.66). It should be noted that the contribution score for a given feature is an aggregated score from all the trees in BoostMEC. For the same sequence feature, its contribution in different sgRNAs may be different as it also depends on other features and the splits they cause in the component trees.

## Discussion

BoostMEC is a novel approach for predicting CRISPR-Cas9 cleavage efficiency, based on a LightGBM learner trained on carefully constructed features. Most existing sgRNA efficiency models rely on one-hot encoding to capture position-specific nucleotide features, resulting in a steep increase in feature dimensions when encoding high-order nucleotides. BoostMEC, however, relies on LightGBM’s ability to directly encode these features as categorical variables. Instead, by creating a sorted histogram for each categorical variable, LightGBM can create efficient splits without constructing deep trees [37]. As with the global feature importance, LightGBM’s holistic handling of categorical variables allows for splits focused on different position-specific mono and di-nucleotides to be grouped together when evaluating impact, allowing for a more comprehensive understanding of the overall importance of each position across the target region, as opposed to importance computed through one-hot encoding.

We note that there are some interesting differences in the feature importance results for BoostMEC and those obtained by Xiang et al. [22] for their alternate CRISPR efficiency prediction model, CRISPRon-GBRT_v1, which, unlike CRISPRon, was trained using gradient boosting regression trees (see Supplementary Note 6 and Supplementary Fig. 12 in [22]). There was a general consensus between the models in terms of the general importance of position-specific sequence values on the 3’ end of the sgRNA, the importance of the 1st nucleotide of the sgRNA, and the strength of the number of TT in the 30-mer region. Nevertheless, compared to BoostMEC, CRISPRon-GBRT_v1 places a much heavier relative importance on thermodynamic features, such as their gRNA-DNA binding energy feature ∆G_B_ (not used in BoostMEC), melting temperature features, and gRNA free energy, as opposed to sequence-based features. Furthermore, for BoostMEC, every position-specific feature in the top 20 was dinucleotide-based, as opposed to CRISPRon-GBRT_v1, which provided (one-hot encoded) a mixed set of mono and dinucleotide position-specific features in its top 20. It is possible that these differences may be due at least in part to how each of these models handles sequence features. One additional difference of note is that only BoostMEC computed the maximum poly-T length and number of poly-T segments, which were also present in the top 20 features for the model.

One advantage of BoostMEC over neural network models is the interpretability of the model, which can be elucidated by feature importance analysis, as well as by additional interpretability tools, such as tree visualizations and LightGBM’s interprete function. These representations allow for further scrutiny of the factors influencing a specific sgRNA’s efficiency or lack thereof. Nevertheless, we acknowledge that there are limits to BoostMEC’s interpretability due to the large number of trees in the model, as well as their depth, both of which add complexity to the model compared to conventional statistical models such as linear regression or generalized linear models. Future research to reduce model complexity is needed for improving interpretability.

Like many other methods, BoostMEC has been developed and tested for editing in U6 and T7 promoter environments using wild-type CRISPR-Cas9, and utilizes information from expanded 30 nt target DNA regions. We found sequence features outside the 20 nt target and PAM region also play important roles. Most pronouncedly, the dinucleotide at position + 1 (di + 1, the first dinucleotide after the PAM site), di -1 (the dinucleotide consisting of the first nucleotide immediately upstream of the 20-mer target and the first nucleotide of the target), di + 2, di −2, di −3 and di −4 ranked as the 15th, 27th, 29th, 31st, 32nd and 33rd most important features in the list (Fig. 4 and Supplementary Table S1 in Additional File 1). Considering the attenuating trend of importance as sequence features get farther away from the seed region, we did not try training BoostMEC with more expanded lengths. Furthermore, BoostMEC has not been tested on other CRISPR systems, such as Cpf1, or on data utilizing other promoter variants, such as U3, nor for non-animal cell types. Future studies and tests are needed to expand the applicability to such variant systems.

## Conclusion

In conclusion, our novel method for the prediction of CRISPR-Cas9 efficiency, BoostMEC, can serve as a powerful and reliable tool for the design and selection of CRISPR-Cas9 sgRNAs in both U6 and T7 promoter environments. Relying on informed feature engineering and boosting (a more conventional machine learning approach as opposed to deep learning), BoostMEC can offer a more informative model showing explicit feature importance as well as more interpretable prediction for any individual sgRNA sequence.

## Methods

### Feature construction

We used two main types of features in the construction of BoostMEC that are all generated from either the expanded target region 30-mer sequence (4 nt context in the 5’ end + 20 nt sgRNA + 3 nt PAM + 3 nt context in the 3’ end) or the 20 nt sgRNA itself. The first category includes the GC content, the frequency of k-mers (for k = 1, 2, 3) in the entire 30 nt expanded target region (4, 16, and 64 features, respectively), the number of poly-T segments (defined as contiguous stretches of 3 or more Ts) in the 30-mer, the length of the longest poly-T segment, and the position dependent k-mer (*k* = 1, 2) instances that may differentiate the sequence motif at different positions relative to the PAM site (28 features each, after removing the static GG from the PAM for each). The second category contains thermodynamic or mechanic metrics derived from the target or sgRNA sequences. The first sub-category contains four melting temperatures calculated from different regions of the 20 nt sgRNA spacer sequence and the first letter of the PAM, including positions 1–21, 1–4, 5–12, and 16–20 (features for these positions termed Tm1, Tm2, Tm3, and Tm4, respectively, as was done in Wang et al. [24]) using the *TmCalculator* R package (version 1.0.1). The second sub-category includes two minimum free energy (∆G) metrics calculated for the 20 nt sgRNA spacer sequence, as well as for the full sgRNA plus the 81 nt WT scaffold sequence using the RNAfold program from the ViennaRNA package [38]. The model in total uses 149 features, utilizing direct encoding of character vectors as opposed to one-hot encoding. More details are available in the Supplementary Materials (Additional File 1).

### BoostMEC model and training

BoostMEC is based on a LightGBM regression model [39] tuned using Bayesian hyperparameter optimization. Optimization was performed using the *rBayesianOptimization* R package (version 1.2.0), with 10 initial sample points, 100 rounds of optimization, and all other settings set to default values. We included a range of model parameters in the tuning process, including the learning rate, maximum tree depth, maximum bin size, maximum number of leaves, and the fraction of columns and rows used in training. The MSE (mean squared error) of each hyperparameter combination was evaluated through tenfold cross-validation on the Kim-Xiang dataset, using the same folds each time. Each model fit had a tree limit of 7,000 and early stopping was employed using the validation fold to prevent overfitting (early_stopping_rounds = 10). The optimal hyperparameters are available in Supplementary Table S4 (Additional File 1). This configuration was then used to train the final BoostMEC model on the entirety of the Kim-Xiang dataset, using the average number of trees obtained in cross-validation.

## Supplementary Information

**Additional file 1: Fig. S1: **Scatter plots comparing BoostMEC predictions and measured efficiency values in test datasets. **Table S1: **LightGBM feature importance values for BoostMEC. **Table S2:** Full prediction interpretation table for GTCTGCCATCTCTGATGGATGTGATGGGCA. **Table S3:** Full prediction interpretation table for GGGGGGACTGTATCGACGCTGAATTGGGGG. **Table S4:** LightGBM hyperparameters.

**Additional file 2: **This file contains a visual representation of BoostMEC’s first regression tree (tree_index = 0).

**Additional file 3: **This file contains a visual representation of BoostMEC’s second regression tree (tree_index = 1).

## Data Availability

The datasets analyzed in this study are freely and publicly available in the supplementary information for Haeussler et al. [25], Kim et al. [21], and Xiang et al. [22], and in the repository for Haeussler et al. [25] https://github.com/maximilianh/crisporPaper. The code for the BoostMEC pipeline, along with demo code, is available at the BoostMEC repository: https://github.com/oazarate/BoostMEC.

## Abbreviations

BoostMEC: Boosting model for efficient CRISPR
CNN: Convolutional neural network
CRISPR: Clustered regularly interspaced short palindromic repeats
gRNA: Guide RNA
LightGBM: Light gradient boosting machine
MSE: Mean squared error
Nt: Nucleotide(s)
PAM: Protospacer adjacent motif
RNA pol III: RNA polymerase III
RNN: Recurrent neural network
sgRNA: Single guide RNA
SVM: Support vector machine
