# Supplementary material for: BoostMEC: predicting CRISPR-Cas9 cleavage efficiency through boosting models
Source: BMC Bioinformatics. 2022 Oct 26;23:446. doi: 10.1186/s12859-022-04998-z (PMC9597963; doi:10.1186/s12859-022-04998-z)
Supplement: Supplementary file 1 — Additional file 1: Fig. S1: Scatter plots comparing BoostMEC predictions and measured efficiency values in test datasets. Table S1: LightGBM feature importance values for BoostMEC. Table S2: Full prediction interpretation table for GTCTGCCATCTCTGATGGATGTGATGGGCA. Table S3: Full prediction interpretation table for GGGGGGACTGTATCGACGCTGAATTGGGGG. Table S4: LightGBM hyperparameters. [file 12859_2022_4998_MOESM1_ESM.docx]

**Supplementary Material**

**BoostMEC: Predicting CRISPR-Cas9 Cleavage Efficiency Through Boosting Models**

Oscar A. Zarate^1^, Yiben Yang^1^, Xiaozhong Wang^2^, Ji-Ping Wang^1,*^

^1^Department of Statistics, Northwestern University, Evanston, IL, USA

^2^Department of Molecular Biosciences, Northwestern University, Evanston, IL, USA

^*^To whom correspondence should be addressed.

**Supplementary Notes: Computing specifications.**

The BoostMEC pipeline is available at <https://github.com/oazarate/BoostMEC>.

A Docker image containing all the required software packages is available on Docker Hub: <https://hub.docker.com/repository/docker/oazarate/boostmec>. Directions on how to use the Docker image for running BoostMEC are available in the BoostMEC GitHub repository.

Analyses for BoostMEC were performed on Linux (Red Hat Enterprise Linux 6), using R version 4.1.0 and Python version 3.7.1.

The Tm_NN() function from the R package TmCalculator (version 1.0.1) was used to calculate DNA melting temperature using default settings.

RNAfold from the ViennaRNA package (version 2.4.14) was used to calculate free energy for sgRNA and sgRNA + scaffold sequences with the settings --noPS -i. The 81 nt scaffold sequence was: GTTTTAGAGCTAGAAATAGCAAGTTAAAATAAGGCTAGTCCGTTATCAACTTGAAAAAGTGGCACCGAGTCGGTGCTTTTT.

The R package for LightGBM (lightgbm version 3.3.2) was used for model training. Both the R package and the Python package (lightgbm == 2.3.1) for LightGBM were used for visualizations.

Predictions for the following models were obtained utilizing the code and instructions from their respective GitHub repositories:

- CRISPRon: <https://github.com/RTH-tools/crispron>
- CRISPRedict: <https://github.com/VKonstantakos/CRISPRedict>
- DeepSpCas9: <https://github.com/MyungjaeSong/Paired-Library>

**Figure S1: Scatter plots comparing BoostMEC predictions and measured efficiency values in test datasets.** This figure shows multiple scatter plots comparing BoostMEC’s predictions and measured efficiency values for sgRNAs from 13 test datasets. Test sequences overlapping with the Kim-Xiang dataset used in the training of BoostMEC were removed. Furthermore, for the xu2015TrainHl60 and xu2015TrainKbm7 datasets, efficiency was measured through log2 fold change in negative selection screens (lower values indicate stronger efficiency), therefore we multiplied the measured efficiency values by -1 for those datasets.


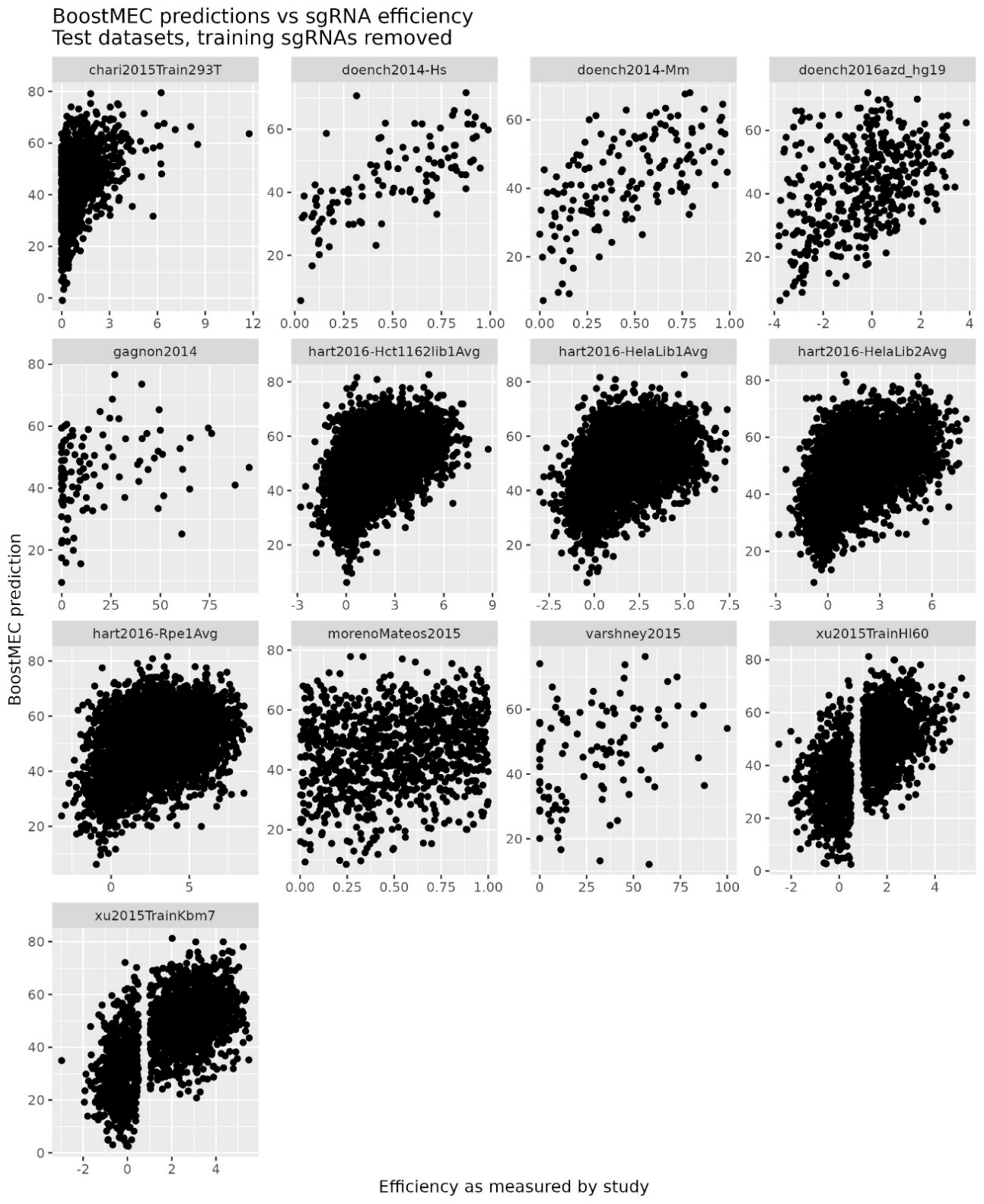


**Table S1: LightGBM feature importance values for BoostMEC.** These feature importance values are sorted by their normalized gain (improvement on the LightGBM objective function) in the BoostMEC model. The normalized cover and frequency, alternative measures of feature importance provided by LightGBM, are also provided here. Position-specific mononucleotides and dinucleotides are specified with a prefix (“mono” or “di”) and the position they pertain to (where the 5’ context, sgRNA and PAM, and 3’ context are specified as positions -4:-1, 1:23, and +1:+3, respectively). Position-independent k-mer counts are represented by each individual k-mer. Tm1, Tm2, Tm3, Tm4 refer to the melting temperatures for positions 1-21, 1-4, 5-12, and 16-20 of the sgRNA + PAM region, modeled after the melting temperature features utilized in Wang et al. (2019).

| **Feature** | **Gain** | **Cover** | **Frequency** |
| --- | --- | --- | --- |
| di 19 | 0.120571 | 0.037055 | 0.033398 |
| di 18 | 0.07912 | 0.038134 | 0.035464 |
| di 17 | 0.062522 | 0.043824 | 0.036957 |
| di 20 | 0.051727 | 0.029792 | 0.027616 |
| tm4 | 0.036838 | 0.014921 | 0.008688 |
| di 16 | 0.032281 | 0.029416 | 0.035791 |
| di 14 | 0.029581 | 0.031299 | 0.038429 |
| TT | 0.027154 | 0.006328 | 0.002876 |
| di 13 | 0.025777 | 0.030154 | 0.031562 |
| di 1 | 0.024576 | 0.027791 | 0.036103 |
| tm1 | 0.024514 | 0.014226 | 0.007216 |
| max_poly_t_len | 0.024507 | 0.015164 | 0.002876 |
| di 15 | 0.023745 | 0.027145 | 0.038644 |
| di 10 | 0.023253 | 0.025881 | 0.034602 |
| di +1 | 0.02285 | 0.027333 | 0.032075 |
| di 3 | 0.021306 | 0.023171 | 0.034958 |
| di 11 | 0.021158 | 0.0231 | 0.036957 |
| grna_energy | 0.019717 | 0.036672 | 0.010226 |
| di 12 | 0.019256 | 0.02728 | 0.033636 |
| num_poly_t | 0.019229 | 0.003313 | 0.00139 |
| di 9 | 0.018948 | 0.024474 | 0.035627 |
| di 4 | 0.017708 | 0.023563 | 0.035471 |
| di 5 | 0.017668 | 0.024052 | 0.033546 |
| di 2 | 0.017658 | 0.024367 | 0.034327 |
| mono 20 | 0.017551 | 0.003178 | 0.001464 |
| di 6 | 0.017195 | 0.022073 | 0.034825 |
| di -1 | 0.016513 | 0.019844 | 0.031904 |
| di 8 | 0.016435 | 0.02425 | 0.035323 |
| di +2 | 0.015909 | 0.025215 | 0.03429 |
| di 7 | 0.014761 | 0.020591 | 0.032632 |
| di -2 | 0.012057 | 0.017014 | 0.030745 |
| di -3 | 0.010435 | 0.015867 | 0.029035 |
| di -4 | 0.010222 | 0.014547 | 0.030039 |
| T | 0.008965 | 0.001781 | 0.002163 |
| gc_count | 0.00864 | 0.007392 | 0.00243 |
| TTT | 0.005932 | 0.006683 | 0.001115 |
| mono 18 | 0.005526 | 0.001777 | 0.001628 |
| mono 19 | 0.00463 | 0.001111 | 0.001308 |
| tm3 | 0.00443 | 0.00758 | 0.004429 |
| grna_scaffold_energy | 0.004071 | 0.019246 | 0.006012 |
| mono +1 | 0.003336 | 0.003232 | 0.001561 |
| AG | 0.003269 | 0.00267 | 0.002044 |
| G | 0.003172 | 0.009158 | 0.002311 |
| mono 17 | 0.002867 | 0.001095 | 0.001471 |
| di 23 | 0.002369 | 0.001851 | 9.59E-04 |
| A | 0.002284 | 0.001175 | 0.001115 |
| mono 1 | 0.002076 | 0.001198 | 0.001271 |
| GGG | 0.002012 | 0.003589 | 0.001516 |
| GG | 0.001527 | 0.007943 | 0.002497 |
| mono 16 | 0.001121 | 4.81E-04 | 7.51E-04 |
| mono 14 | 0.001089 | 5.06E-04 | 7.36E-04 |
| CTT | 8.43E-04 | 0.00231 | 9.22E-04 |
| CCA | 7.68E-04 | 4.60E-04 | 0.001293 |
| tm2 | 7.34E-04 | 0.005003 | 0.002512 |
| GCC | 7.33E-04 | 0.002559 | 0.001211 |
| ATT | 7.33E-04 | 0.002955 | 7.28E-04 |
| CAG | 7.32E-04 | 6.53E-04 | 9.59E-04 |
| TCC | 7.00E-04 | 0.013007 | 0.001999 |
| TAC | 6.98E-04 | 0.005241 | 8.55E-04 |
| GA | 6.97E-04 | 3.60E-04 | 8.17E-04 |
| mono 21 | 5.61E-04 | 7.88E-04 | 8.40E-04 |
| CA | 5.55E-04 | 0.006652 | 0.001494 |
| TC | 5.28E-04 | 0.004185 | 0.00107 |
| GAG | 4.94E-04 | 2.07E-04 | 5.20E-04 |
| CGG | 4.42E-04 | 0.010948 | 0.001546 |
| mono 10 | 4.17E-04 | 4.10E-04 | 6.69E-04 |
| AAA | 4.11E-04 | 0.005498 | 8.32E-04 |
| TTC | 3.17E-04 | 1.48E-04 | 2.75E-04 |
| CG | 3.07E-04 | 0.001024 | 7.36E-04 |
| mono 11 | 2.98E-04 | 2.07E-04 | 4.01E-04 |
| AC | 2.94E-04 | 6.56E-04 | 5.87E-04 |
| C | 2.91E-04 | 0.001956 | 8.84E-04 |
| di 21 | 2.87E-04 | 3.56E-04 | 4.01E-04 |
| TTG | 2.84E-04 | 1.72E-04 | 2.45E-04 |
| mono +3 | 2.79E-04 | 3.33E-04 | 5.72E-04 |
| TA | 2.53E-04 | 2.41E-04 | 5.35E-04 |
| CC | 2.52E-04 | 0.003699 | 7.65E-04 |
| AGA | 2.50E-04 | 1.10E-04 | 3.20E-04 |
| mono 6 | 2.44E-04 | 2.18E-04 | 4.53E-04 |
| GTA | 2.43E-04 | 0.002417 | 5.13E-04 |
| GGA | 2.32E-04 | 0.002637 | 6.17E-04 |
| TCT | 2.24E-04 | 7.90E-05 | 2.30E-04 |
| TGG | 2.09E-04 | 0.009732 | 0.001241 |
| CCG | 2.03E-04 | 0.004651 | 8.55E-04 |
| mono 3 | 1.94E-04 | 1.47E-04 | 3.34E-04 |
| AGC | 1.75E-04 | 2.57E-04 | 4.24E-04 |
| CCC | 1.65E-04 | 0.001264 | 3.94E-04 |
| mono 15 | 1.62E-04 | 1.42E-04 | 3.49E-04 |
| CT | 1.57E-04 | 2.48E-04 | 3.34E-04 |
| ATG | 1.57E-04 | 0.003232 | 5.72E-04 |
| mono 9 | 1.56E-04 | 2.04E-04 | 4.24E-04 |
| GTC | 1.56E-04 | 0.004122 | 6.39E-04 |
| ACC | 1.50E-04 | 9.42E-04 | 3.42E-04 |
| GCA | 1.50E-04 | 1.07E-04 | 3.57E-04 |
| GGC | 1.48E-04 | 0.002636 | 4.76E-04 |
| TCG | 1.45E-04 | 0.002481 | 4.61E-04 |
| mono 13 | 1.34E-04 | 1.28E-04 | 3.34E-04 |
| TGC | 1.34E-04 | 0.001531 | 4.98E-04 |
| GGT | 1.34E-04 | 0.001062 | 4.31E-04 |
| ACA | 1.30E-04 | 0.003617 | 5.57E-04 |
| mono 4 | 1.26E-04 | 8.73E-05 | 2.30E-04 |
| GAA | 1.23E-04 | 0.002653 | 5.05E-04 |
| AAG | 1.21E-04 | 0.001087 | 3.94E-04 |
| mono 12 | 1.15E-04 | 9.63E-05 | 2.90E-04 |
| GTG | 1.11E-04 | 1.02E-04 | 2.38E-04 |
| GCT | 1.07E-04 | 0.002475 | 5.05E-04 |
| CCT | 1.05E-04 | 1.54E-04 | 2.75E-04 |
| GC | 1.02E-04 | 0.002395 | 4.83E-04 |
| TG | 9.71E-05 | 2.04E-04 | 2.82E-04 |
| GTT | 9.70E-05 | 0.005372 | 6.17E-04 |
| AGG | 9.48E-05 | 9.65E-05 | 2.01E-04 |
| AT | 9.03E-05 | 0.004498 | 6.17E-04 |
| GT | 9.01E-05 | 0.002202 | 4.61E-04 |
| TTA | 8.32E-05 | 0.002328 | 3.12E-04 |
| AA | 8.18E-05 | 0.001075 | 3.20E-04 |
| mono +2 | 8.00E-05 | 6.26E-05 | 2.08E-04 |
| mono 5 | 7.66E-05 | 1.02E-04 | 2.53E-04 |
| CTC | 7.43E-05 | 0.001076 | 3.12E-04 |
| TCA | 7.35E-05 | 4.57E-04 | 2.16E-04 |
| CTG | 7.16E-05 | 8.60E-05 | 1.93E-04 |
| mono 8 | 6.55E-05 | 9.37E-05 | 2.01E-04 |
| mono -1 | 6.36E-05 | 1.24E-04 | 2.60E-04 |
| GAC | 6.33E-05 | 6.27E-05 | 1.56E-04 |
| mono 2 | 6.31E-05 | 6.71E-05 | 2.01E-04 |
| CGT | 6.26E-05 | 7.34E-04 | 2.16E-04 |
| CGA | 5.98E-05 | 0.00417 | 5.13E-04 |
| mono 7 | 5.44E-05 | 8.05E-05 | 2.01E-04 |
| AAT | 4.94E-05 | 4.11E-04 | 1.26E-04 |
| GCG | 4.90E-05 | 2.70E-04 | 1.34E-04 |
| mono -4 | 4.49E-05 | 9.23E-05 | 2.01E-04 |
| TGA | 4.30E-05 | 1.41E-04 | 1.63E-04 |
| ACT | 4.18E-05 | 1.03E-04 | 1.11E-04 |
| CAT | 3.89E-05 | 5.78E-05 | 1.34E-04 |
| mono -2 | 3.87E-05 | 3.95E-05 | 1.34E-04 |
| ACG | 3.59E-05 | 1.54E-04 | 7.43E-05 |
| TAG | 3.35E-05 | 8.36E-04 | 1.63E-04 |
| CGC | 3.27E-05 | 0.001126 | 2.08E-04 |
| CAC | 3.10E-05 | 3.78E-05 | 1.11E-04 |
| mono -3 | 3.07E-05 | 4.39E-05 | 1.49E-04 |
| AAC | 2.66E-05 | 0.001173 | 2.01E-04 |
| TGT | 2.55E-05 | 4.62E-05 | 9.66E-05 |
| TAA | 2.17E-05 | 9.62E-04 | 1.34E-04 |
| AGT | 2.04E-05 | 3.59E-05 | 5.95E-05 |
| CTA | 1.90E-05 | 7.04E-05 | 5.20E-05 |
| CAA | 1.35E-05 | 2.69E-05 | 4.46E-05 |
| GAT | 1.21E-05 | 1.50E-05 | 3.72E-05 |
| ATC | 1.13E-05 | 3.20E-05 | 5.20E-05 |
| ATA | 8.50E-06 | 8.29E-04 | 9.66E-05 |
| TAT | 5.73E-06 | 5.46E-05 | 2.97E-05 |

**Table S2. Full prediction interpretation table for GTCTGCCATCTCTGATGGATGTGATGGGCA.** This table provides the complete feature contribution values for the BoostMEC efficiency prediction (49.53) for sgRNA GTCT-GCCATCTCTGATGGATGTGA-TGG-GCA from the doench2014-Hs dataset. The top 10 values from this table are plotted in Figure 6A in the main text.

| **Feature** | **Contribution** |
| --- | --- |
| di 19 | 3.976647 |
| di +1 | -2.50713 |
| di 10 | 2.298254 |
| di 17 | 2.084711 |
| di 18 | 1.761352 |
| di 20 | -1.63808 |
| di 13 | -1.44552 |
| mono +1 | -1.17263 |
| di 5 | 1.007038 |
| di 3 | -0.99935 |
| di 4 | 0.989531 |
| di 7 | -0.9281 |
| di -1 | 0.889853 |
| grna_energy | -0.81384 |
| TT | 0.813154 |
| di 14 | 0.755161 |
| AG | -0.68291 |
| di 16 | 0.674805 |
| di 12 | 0.640224 |
| di 8 | 0.562534 |
| max_poly_t_len | 0.524221 |
| di 23 | -0.52085 |
| num_poly_t | 0.511475 |
| di 1 | 0.479287 |
| di 2 | 0.477063 |
| tm4 | -0.44632 |
| di 11 | 0.405105 |
| di 15 | -0.3298 |
| di 9 | -0.3252 |
| TC | -0.30916 |
| tm1 | 0.291543 |
| grna_scaffold_energy | 0.290689 |
| di 6 | 0.2685 |
| mono 18 | -0.26505 |
| di -2 | 0.257909 |
| T | -0.24465 |
| AT | 0.216193 |
| TTT | 0.198842 |
| mono 1 | 0.181679 |
| mono 19 | 0.181026 |
| mono 21 | -0.15625 |
| TAC | -0.12155 |
| TCC | -0.11629 |
| CA | 0.115431 |
| GTC | -0.10722 |
| ATT | 0.106941 |
| CGG | -0.1065 |
| AAA | 0.102412 |
| di -3 | -0.10137 |
| mono 14 | -0.09842 |
| di +2 | 0.097792 |
| GGG | -0.0962 |
| mono 16 | -0.08574 |
| di 21 | -0.08287 |
| ATG | 0.067942 |
| TGG | 0.065827 |
| mono 20 | -0.06232 |
| GTA | -0.05727 |
| tm2 | -0.05581 |
| gc_count | 0.055661 |
| TCG | 0.053015 |
| GCT | -0.0504 |
| A | 0.049345 |
| CCG | -0.04829 |
| CTT | 0.048143 |
| GTT | -0.04508 |
| tm3 | -0.04398 |
| mono 12 | -0.03965 |
| di -4 | -0.03929 |
| mono 11 | 0.037955 |
| GGA | -0.03625 |
| TTG | 0.035695 |
| CC | 0.033271 |
| TGA | -0.03275 |
| GG | -0.03197 |
| CCT | 0.031779 |
| mono 17 | 0.030973 |
| CTC | 0.029314 |
| mono +3 | -0.0279 |
| TA | -0.02584 |
| CAG | -0.02572 |
| mono 3 | -0.02277 |
| G | 0.021006 |
| GAA | -0.01974 |
| CG | -0.01741 |
| TG | 0.016911 |
| TTC | 0.016279 |
| mono +2 | -0.01549 |
| GAG | -0.01543 |
| ACC | -0.01476 |
| TGT | -0.01362 |
| mono 8 | 0.013429 |
| mono -4 | 0.012748 |
| GGC | -0.01237 |
| CGA | 0.012108 |
| mono 15 | 0.011518 |
| mono 10 | -0.01151 |
| ACA | -0.01086 |
| AC | -0.01077 |
| CCA | -0.01009 |
| CGT | 0.009793 |
| mono 7 | -0.00977 |
| mono 9 | -0.00963 |
| mono 13 | -0.00898 |
| CGC | -0.0081 |
| GCA | 0.00809 |
| TAG | 0.007858 |
| CAT | 0.007693 |
| TTA | 0.006764 |
| mono -1 | 0.006646 |
| mono 5 | -0.00624 |
| CCC | 0.005858 |
| GCC | 0.005654 |
| TGC | 0.005651 |
| mono 6 | 0.005456 |
| AAG | -0.00543 |
| AAC | -0.00516 |
| GGT | 0.004924 |
| C | 0.004844 |
| GA | 0.004604 |
| CTA | -0.00445 |
| CT | -0.00389 |
| GT | -0.00367 |
| ATA | -0.00366 |
| GC | -0.00354 |
| TAA | -0.00341 |
| AGG | 0.003348 |
| ACT | 0.002345 |
| AAT | 0.002197 |
| AA | 0.001911 |
| GTG | 0.001444 |
| ACG | 0.001361 |
| GCG | -0.00122 |
| AGC | -0.00104 |
| TAT | -6.94E-04 |
| TCA | -1.50E-04 |

**Table S3. Full prediction interpretation table for GGGGGGACTGTATCGACGCTGAATTGGGGG.** This table provides the complete feature contribution values for the BoostMEC efficiency prediction (28.42) for sgRNA GGGG-GGACTGTATCGACGCTGAAT-TGG-GGG from the morenoMateos2015 dataset. The top 10 values from this table are plotted in Figure 6B in the main text.

| **Feature** | **Contribution** |
| --- | --- |
| di 18 | -6.66026 |
| di 19 | -4.19574 |
| tm4 | -3.38042 |
| di 20 | -2.58258 |
| di +1 | -2.27593 |
| di 15 | -1.86618 |
| di 17 | 1.841555 |
| di 14 | -1.81298 |
| di 1 | 1.777987 |
| di 13 | -1.71384 |
| di 2 | 1.454986 |
| di 11 | 1.238753 |
| di 6 | 1.203107 |
| di 12 | 0.971162 |
| di 16 | 0.934906 |
| AG | -0.87211 |
| T | 0.840482 |
| num_poly_t | 0.813671 |
| di 4 | -0.8026 |
| di 3 | 0.722886 |
| di 7 | 0.709146 |
| grna_energy | 0.696816 |
| di 10 | -0.67244 |
| di 5 | 0.650393 |
| TT | 0.631868 |
| mono +1 | -0.58157 |
| GGG | -0.57838 |
| di -1 | 0.50978 |
| max_poly_t_len | 0.504261 |
| mono 18 | -0.47683 |
| mono 20 | -0.40797 |
| di +2 | -0.36832 |
| GG | 0.368241 |
| di 23 | -0.3343 |
| tm1 | 0.315015 |
| di 9 | -0.30298 |
| di -3 | -0.28811 |
| ATT | -0.28426 |
| mono 17 | 0.264031 |
| gc_count | 0.226196 |
| GTA | 0.191471 |
| mono 21 | -0.17947 |
| GA | 0.172001 |
| mono 1 | 0.170588 |
| mono 19 | -0.1694 |
| TCC | -0.16012 |
| mono 16 | -0.14891 |
| grna_scaffold_energy | 0.140954 |
| TTT | 0.134912 |
| TAC | -0.12175 |
| di -4 | 0.111162 |
| TC | 0.095619 |
| CTT | 0.092546 |
| AAA | 0.086927 |
| G | 0.086788 |
| TCG | -0.07796 |
| di -2 | 0.075836 |
| GCC | 0.074683 |
| CGG | -0.07187 |
| CCA | -0.06954 |
| CAG | -0.06825 |
| di 21 | -0.06359 |
| mono 11 | 0.060612 |
| CA | 0.056651 |
| GTC | 0.055735 |
| TTG | -0.05311 |
| GCT | 0.04708 |
| di 8 | -0.04687 |
| GGT | -0.042 |
| GTT | -0.04095 |
| mono 13 | -0.03922 |
| AAT | -0.03805 |
| C | 0.037869 |
| mono 10 | -0.03668 |
| ATG | -0.0364 |
| mono 3 | 0.033728 |
| tm2 | -0.03346 |
| AAG | -0.03169 |
| AGA | -0.03014 |
| CGA | -0.02934 |
| A | -0.02863 |
| GGA | -0.02848 |
| CCG | -0.02339 |
| AT | -0.02211 |
| TTC | 0.017988 |
| TGG | -0.0179 |
| mono +3 | 0.017834 |
| mono 6 | 0.017291 |
| mono -4 | 0.016905 |
| CGC | 0.016798 |
| mono 12 | 0.016468 |
| mono -1 | -0.01592 |
| mono 4 | -0.01541 |
| CTC | -0.01344 |
| TTA | 0.013237 |
| TGC | 0.013092 |
| AGG | -0.01298 |
| ACT | -0.01251 |
| mono 8 | 0.012214 |
| ACA | -0.01133 |
| GAA | -0.01123 |
| CCC | -0.01101 |
| CAT | 0.01089 |
| mono 9 | -0.01043 |
| GGC | -0.01023 |
| TA | 0.010191 |
| CGT | 0.009084 |
| GC | -0.00875 |
| GCG | 0.008474 |
| GAG | -0.00825 |
| CC | -0.00745 |
| ACC | -0.00744 |
| AAC | -0.00653 |
| GT | -0.00615 |
| CT | -0.00595 |
| ATA | -0.00585 |
| AGC | -0.00571 |
| GCA | -0.00559 |
| TAT | 0.005446 |
| TG | -0.00535 |
| TAG | 0.005037 |
| mono 2 | -0.00448 |
| ACG | -0.00382 |
| TAA | -0.00346 |
| CTA | -0.0034 |
| TGA | 0.003362 |
| CG | -0.00333 |
| tm3 | -0.00313 |
| AC | -0.00278 |
| AA | -0.00218 |
| TCA | 0.001605 |
| mono 14 | -5.13E-04 |

**Table S4: LightGBM hyperparameters.** Hyperparameter optimization was carried out using the *rBayesianOptimization* package with 10 initial sample points, 100 rounds of optimization, and all other settings set to default values. Within that optimization process, candidate hyperparameter combinations were evaluated via 10-fold cross-validation with early stopping using the hold-out fold (early_stopping_rounds = 10). An upper limit of 7,000 trees was set. The following parameters were held fixed: objective = regression, boosting = gbdt. Furthermore, we trained using 14 cores and set num_threads = 14.

| **Parameter** | **Candidate value range** | **Optimal set** |
| --- | --- | --- |
| learning_rate | 0.001 – 0.2 | 0.009896071 |
| min_data | 0 – 300 | 203 |
| max_bin | 50 – 500 | 343 |
| max_depth | -1 – 20 (-1 removes limit) | 12 |
| num_leaves | 2 – 1000 | 701 |
| feature_fraction | 0.1 – 1 | 0.4832467 |
| bagging_fraction | 0.1 – 1 | 0.1345159 |
| lambda_l2 | 0 – 10 | 1.482597 |
