# Supplementary material for: BoostMEC: predicting CRISPR-Cas9 cleavage efficiency through boosting models
Source: BMC Bioinformatics. 2022 Oct 26;23:446. doi: 10.1186/s12859-022-04998-z (PMC9597963; doi:10.1186/s12859-022-04998-z)
Supplement: Supplementary file 2 — Additional file 2: This file contains a visual representation of BoostMEC’s first regression tree (tree_index = 0). [file 12859_2022_4998_MOESM2_ESM.pdf]

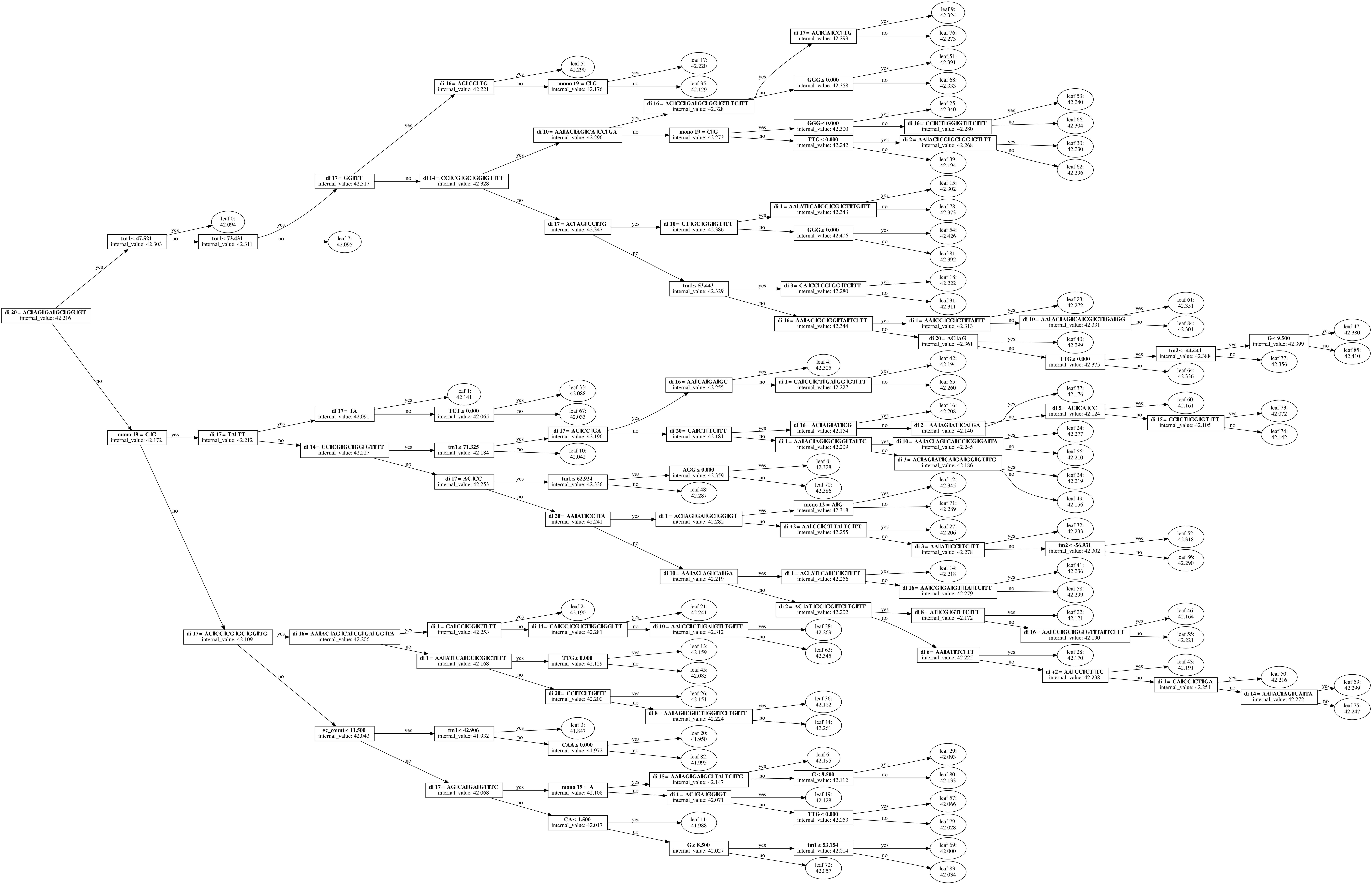

BoostMEC_tree_0: Schematic plot of regression trees from BoostMEC. BoostMEC consists of many sequencing trees. Plotted is the first (tree index=0) regression trees from BoostMEC (partial tree was plotted in Figure 6A). The tree starts with the grand mean of the efficiency score (internal_value = 42.216) and splits based on whether the dinucleotide at position 20 is one of several values (di 20 = AC||AG||GA||GC||GG||GT) and so forth. The internal_value at each node represents the mean within each branch under the mean squared error loss function. An oval shape represents an end node whereas a rectangle represents an intermediate node that grows further until reaching leaf nodes.
