# Supplementary figures and images for: BoostMEC: predicting CRISPR-Cas9 cleavage efficiency through boosting models
Source: BMC Bioinformatics. 2022 Oct 26;23:446. doi: 10.1186/s12859-022-04998-z (PMC9597963; doi:10.1186/s12859-022-04998-z)

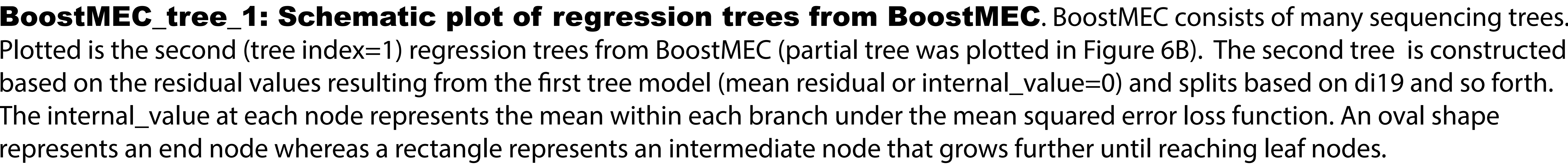

Supplement: Supplementary file 3 — Additional file 3: This file contains a visual representation of BoostMEC’s second regression tree (tree_index = 1). [file 12859_2022_4998_MOESM3_ESM.pdf]
